# Plant, space and time - linked together in an integrative and scalable data management system for phenomic approaches in agronomic field trials

**DOI:** 10.1186/s13007-020-00596-3

**Published:** 2020-04-21

**Authors:** Andreas Honecker, Henrik Schumann, Diana Becirevic, Lasse Klingbeil, Kai Volland, Steffi Forberig, Marc Jansen, Hinrich Paulsen, Heiner Kuhlmann, Jens Léon

**Affiliations:** 1 INRES-Plant Breeding, University of Bonn, Katzenburgweg 5, 53115 Bonn, Germany; 2 IGG-Geodesy, University of Bonn, Nussallee 17, 53115 Bonn, Germany; 3 Terrestris GmbH & Co. KG, Kölnstraße 99, 53111 Bonn, Germany

**Keywords:** Phenotyping, Field trials, Crop plants, Soil, Weather, Data management system, Geo-coordinates, Timestamps, WebGIS, Open source

## Abstract

**Background:**

To ensure further genetic gain, genomic approaches in plant breeding rely on precise phenotypic data, describing plant structure, function and performance. A more precise characterization of the environment will allow a better dealing with genotype-by-environment-by-management interactions. Therefore, space and time dependencies of the crop production processes have to be considered. The use of novel sensor technologies has drastically increased the amount and diversity of phenotypic data from agronomic field trials. Existing data management systems either do not consider space and time, are not customizable to individual needs such as field trial handling, or have restricted availability. Hence, we propose an integrative data management and information system (DMIS) for handling of traditional and novel sensor-based phenotypic, environmental and management data. The DMIS must be customizable, applicable and scalable from individual users to organizations.

**Results:**

Key element of the system is a dynamic PostgreSQL database with GIS-extension, capable of importing, storing and managing all types of data including images. The database references every structural database object and measurement in a threefold approach with semantic, spatial and temporal reference. Timestamps and geo-coordinates allow automated linking of all data. Traits can be precisely defined individually or uploaded as predefined lists. Filtering and selection routines allow compilation of all data for visualization via tables, charts or maps and for export and external statistical analysis. New possibilities of environmental information-based planning of field trials, weather-guided phenotyping and data analysis for outlier or hot-spot detection are demonstrated.

**Conclusions:**

The DMIS supports users in handling experimental field trials with crop plants and modern phenotyping methods. It focuses on linking all space and time dependent processes of plant production. Weather, soil and management, as well as growth and yield formation of the plants can be depicted, thus allowing a more precise interpretation of the results in relation to environment and management. Breeders, extension specialists, official testing agencies and agricultural scientists are assisted in all steps of a typical workflow with planning, designing, conducting, controlling and analyzing field trials to generate new information for decision support in the crop improvement process.

## Background

Plant breeders always collected phenotypic information, mostly by scoring and measuring traits, for the selection of the most promising candidates. Modern phenotyping must provide precise information on plant structure, physiological functions and performance [1]. This data can also be used to train the models for genomic prediction [2]. Physiology-related processes and plant growth are dependent on the permanently changing environmental conditions in field. Therefore, data characterizing these environmental conditions, sensed by plants and plant organs at temporal scales ranging from minutes (for metabolism and hydraulics) to months (for yield), is needed to properly analyze phenotypic datasets [1].

Within a field, several factors interact, generating microenvironments that differ from plot to plot, influencing several traits including yield. Since spatial variation is commonly known in agricultural field trials, it is important to correct for these factors when estimating genotypic effects [3]. Thus, bias of genotypic effects can be reduced and the resulting accuracy can be increased [4]. Approaches to account for this variation are grid soil sampling [5], electromagnetic soil mapping [6] and soil water and temperature sensor networks [7]. In general, a more precise characterization of the environment (“Envirotyping”) is requested [8]. Aims are a better understanding of the genotype-by-environment-by-management interactions [9] and the stresses under the target conditions [10]. Improved methods to analyze the data and to model the differences in plant behavior between experiments in “field multi-environment networks” might become possible [1].

Based on the recent development of novel sensor technologies, new non-invasive image-based phenotyping methods are available at plant and canopy level, allowing non-destructive crop monitoring over time. They provide first insights into the physiology of crops [11, 12]. New geometric traits for crop canopies, like plant height or lodging area, can automatically be extracted from multiple images using unmanned aerial vehicles (UAVs) [13–15]. Time-series of these non-invasive measurements during vegetation can link phenotyping and crop modeling [1].

As a result, availability, quantity and heterogeneity of phenotypic data have increased significantly over the past decade [16, 17]. It is requested, that new data management tools need to be integrated into the phenotyping pipelines to handle this data [18]. Araus et al. conclude, to translate high-throughput field phenotyping into genetic gain, “it also requires appropriate trial management and spatial variability handling, definition of key constraining conditions prevalent in the target population of environments, and the development of more comprehensive data management” [14].

In the context of crop improvement, this conclusion likewise applies to plant scientists searching for new physiological traits, or proxies of traits, to dissect complex breeding targets like drought tolerance [19, 20]. It is also relevant to commercial breeding companies, governmental agencies and organizations conducting multi-environment field trials for variety registration and agronomic extension. However, requirements for the data management systems are user-specific and heterogeneous. This ranges from substituting pen and paper by personal digital assistants (PDA) in scoring and research documentation [21], to establishing solutions for transferring spreadsheet format to databases [22], to visions about scale-independent meta-analyzes on large phenomic datasets [1]. Analysis of the published data management systems and commercial solutions shows great heterogeneity in terms of scope, functionality and accessibility.

Large scale data management systems of national research collaborations such as the “Phenotyping Hybrid Information System” [23] or the “Brassica Information Portal” [24] were proposed in the academic field. These systems handle phenotypic and genomic data by collecting them in a central database and making it accessible to users within the community. Data gets structurally stored via ontologies, linking experiments as well as crop- and environmental traits. While the Brassica Information Portal is specifically designed for Brassica species, the Phenotyping Hybrid Information System is not crop specific.

The system “CropSight” [25] uses internet-of-things-approaches to automate the capture and storage of phenotypic and environmental data. Based on intelligent sensors and mobile smart devices, it comes with initial costs for infrastructure establishment. The software “Plabsoft” [26] is described primarily as a database developed for breeding experiments. It allows linking of phenotypic traits and genomic information and performing statistical analysis due to a connection to the R software package [27]. Addressing the collection of phenotypic data, the software tool “Phenotyper” [21] allows to import and manage phenotypic data using PDAs. The user can store data directly in the central database “Phenotyper” [28]. A definition of traits and structures is implemented and defined formats from given ontologies enable the structural storage of phenotypic data. According to our knowledge geospatial information has not been considered so far.

The proprietary software “MiniGis” [29] is designed for commercial breeders including options to create experimental designs with attached geographic (geo) coordinates. Field trials can virtually be placed on the field and plots can subsequently be sown with support of global navigation satellite systems such as the global positioning system (GPS). Import of measurement data and their storage is not possible in the basic application. In official variety and seed testing trials in Germany, data management is mostly done with the commercial software “PIAF” [30]. It addresses the structural organization of field trials through experimental factors to serve as a tool for planning, data collection and analysis of field trial data. Geo-spatial information has not yet been considered.

Existing commercial farm management and information systems are generally personalized and tailored to single users [31, 32]. They concentrate on solving and documenting daily farm tasks, e.g. management of field operations, machinery or human resources. While these systems mostly include geo-referencing of data, they lack the capacity for specific data collection, similar to phenotyping, or field trials with structured experimental factors.

Concluding, the aim of the project was a flexible, integrative data management and information system (DMIS). It should be capable of representing field trial scenarios with data from traditional and image-based phenotyping, as well as from environment and agricultural management. The use of geo-coordinates and timestamps for all structural database objects (entities) and measurements should enable to linking these data focusing on the time and space dependent growth and yield formation of the plants. An open system architecture and scalability should make it applicable for every user in the field of crop improvement.

## Implementation

### System architecture of the DMIS

**Fig. 1:**
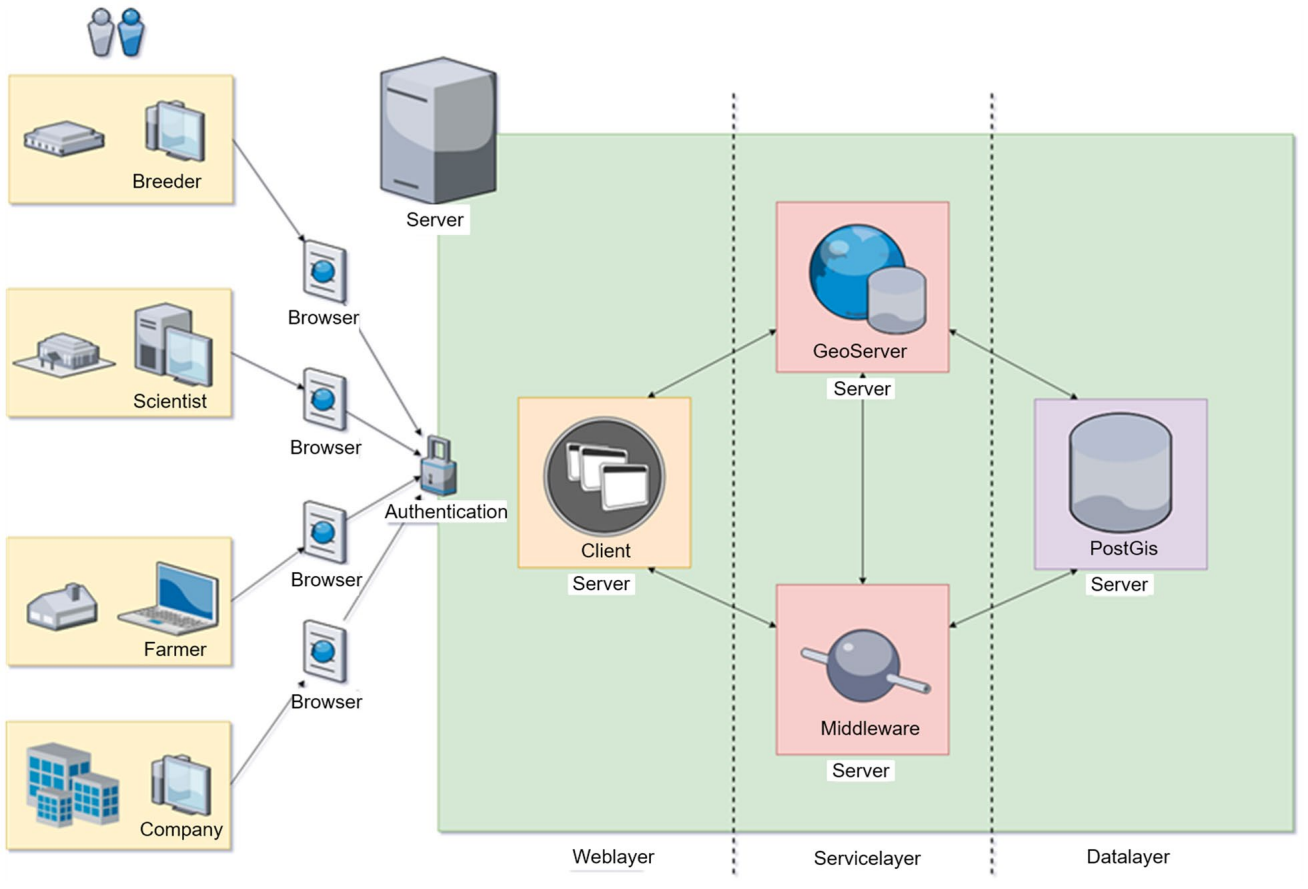
System architecture of the DMIS. Users authenticate and access the application on the server via browsers. The system components are installed in Dockers and communicate with each other via HTTP. The GUI (Client) is localized in the Weblayer, the PostgreSQL and PostGIS database components in the Datalayer, the GeoServer and a Middleware are acting in the Servicelayer, connecting the client application and the database

The system architecture of the data management and information system is designed to run on local servers or in the cloud (Fig. 1). After authentication, users can access the application via a browser-based graphical user interface (GUI). This protects the application and the data from unauthorized access. Since browsers are used, an online or offline network link is required to operate the system. In the case of an online network link, the server must be accessible on the internet. Likewise, it is possible to run the application in an intranet. Server and user then need connections to this externally closed network.

### Software and database

The key component within the data management and information system is a dynamic PostgreSQL database with PostGIS extender, referred to as Central Processing and Exchange Database (CPED), as illustrated in the Datalayer (Fig. 1). The software is programmed in JavaScript and licensed by the BSD 2-Clause License. There are no restrictions to non-academic use. It is capable of storing all numerical and non-numerical data occurring in the plant production process. Image data acquired is referenced within the database, but separately stored within a folder structure. Automated backups support secure data storage. The GeoServer does the spatial referencing via geo-coordinates and the Middleware mediates between the software components.

The web application has been completely created with OpenSource software and the most important software libraries used are react-geo [33], OpenLayers [34], D3 [35], AG-Grid [36], GeoStyler [37], sequelize [38] and express [39].

### Hardware

The system is based on docker containers. These virtual containers contain all packages needed to run and therefore can be run on any server which supports docker [40]. For reliable performance of the system, server specifications of 8 GB of RAM and a multicore CPU with at least 3 GHz are recommended. The web-application can be accessed by any connected smart device capable of running standard web-browsers.

## Results

### Field trials as origin of data

Basis was a two-year field trial with 12 winter wheat varieties in two management systems at two testing sites within four replications. The two cropping systems allowed tracking of differing field management, while the two locations and 2 years represented four different environments. As environmental information, soil characteristics were determined by soil sampling and electromagnetic soil mapping (EM38 [41]) and weather parameter in atmosphere and soil were monitored by weather stations in all fields. Besides counting, measuring and sampling for determining traditional plant traits, every 2 weeks digital imaging of the crop stands was done by UAVs (20–30 m canopy distance) and a tractor based pheno-mobile (two m canopy distance). Using image-analysis-pipelines, plant parameters like plant height or vital leaf area of plants were extracted. Management measures, yield and quality parameters were recorded. Raw and processed data, as well as raw and processed images (e.g. orthophotos), were referenced by space and time and imported to the system.

### Components of the data management and information system

**Fig. 2:**
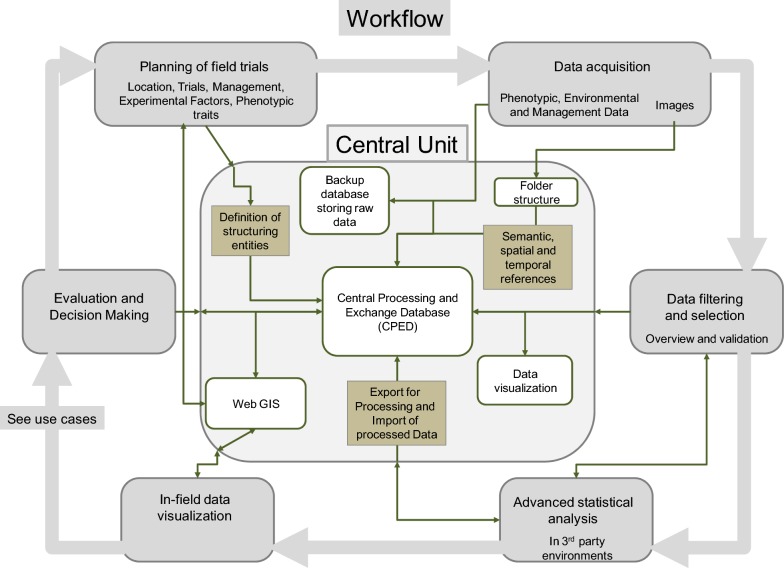
Components of the Central Unit and their use in an exemplary workflow of an agronomic field trial. The Central Unit consists of a central processing and exchange database (CPED) and a backup database. Images are stored in a folder structure and referenced in the CPED. Structuring database objects (entities) e.g. farm, field or plot, can be defined according to the experiment. Data can be imported, filtered, combined, visualized and exported. Data visualization tools and a WebGIS are implemented and can be used to assist in final evaluation and decision-making

The DMIS consists of a Central Unit (Fig. 2), that can be set up according to the users’ needs, either on single PCs, local servers, or in the cloud. Users can interact with the DMIS via web browsers. A GUI allows the realization of all steps within a typical workflow of an agronomic field trial.

**Fig. 3:**
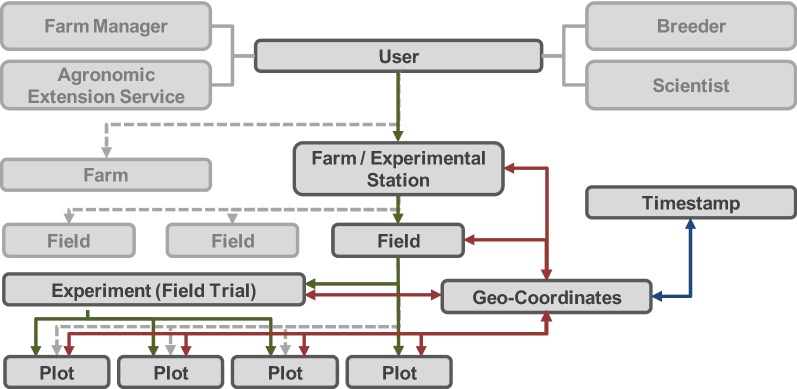
Semantic referencing scheme with structuring objects (entities) within the DMIS. Database structure relies on a three-fold referencing scheme with semantic (green), spatial (red) and temporal (blue) references. Solid lines indicate relations in terms of an experimental field trial. Dashed lines represent available options to depict single or multiple farm structures as demanded by different users. 3-fold referencing allows the integration of all available data within the plant production process

The CPED structures, stores and manages all data. When importing numerical data, data is stored in the CPED and automatically mirrored to a backup database. Thus, the storage of valuable raw data is guaranteed without being altered or processed by users. Due to the large file size, images are not stored in the CPED, but saved in a folder structure and referenced by geo-coordinates and time. Images can be linked to fields, trials and plots by their spatial and temporal reference, or connected due to the semantic structure (Fig. 3). The geo-referencing allows the integrated geographic information system (WebGIS) to access and utilize the stored data. A technical link to publicly available data via web map services (WMS) opens additional opportunities [42]. Data such as soil maps, aerial, or satellite images can be imported as layers and used as further information. The implemented data visualization tools allow a direct illustration of the imported data.

To manage all relevant data from the plant production process, the database structure refers to a semantic scheme from individual users to spatial structures (Fig. 3). Farms, fields, plots and field trials with specified experimental factors can be defined as structuring database objects (entities) and weather stations can be integrated. For users not conducting field trials, like farmers, plots are directly attached to fields. The DMIS defines all structuring entities spatially via geo-coordinates and relates them to geographical structures. An additional temporal reference is realized by attaching timestamps to entities and measurements. Concluding, all sources of data can either be semantically connected to a farm, field, plot or experimental trial, or directly linked and accessed by its spatial and temporal properties. Besides this schematic illustration, a detailed visualization of the data model is given in (Additional file 1).

### Graphical user interface and realized workflow

Within the GUI, all steps of the workflow of an agronomic field trial from initial planning to the deduction of information to support decisions can be performed as an interaction between the user and the DMIS via web browsers.

**Fig. 4:**
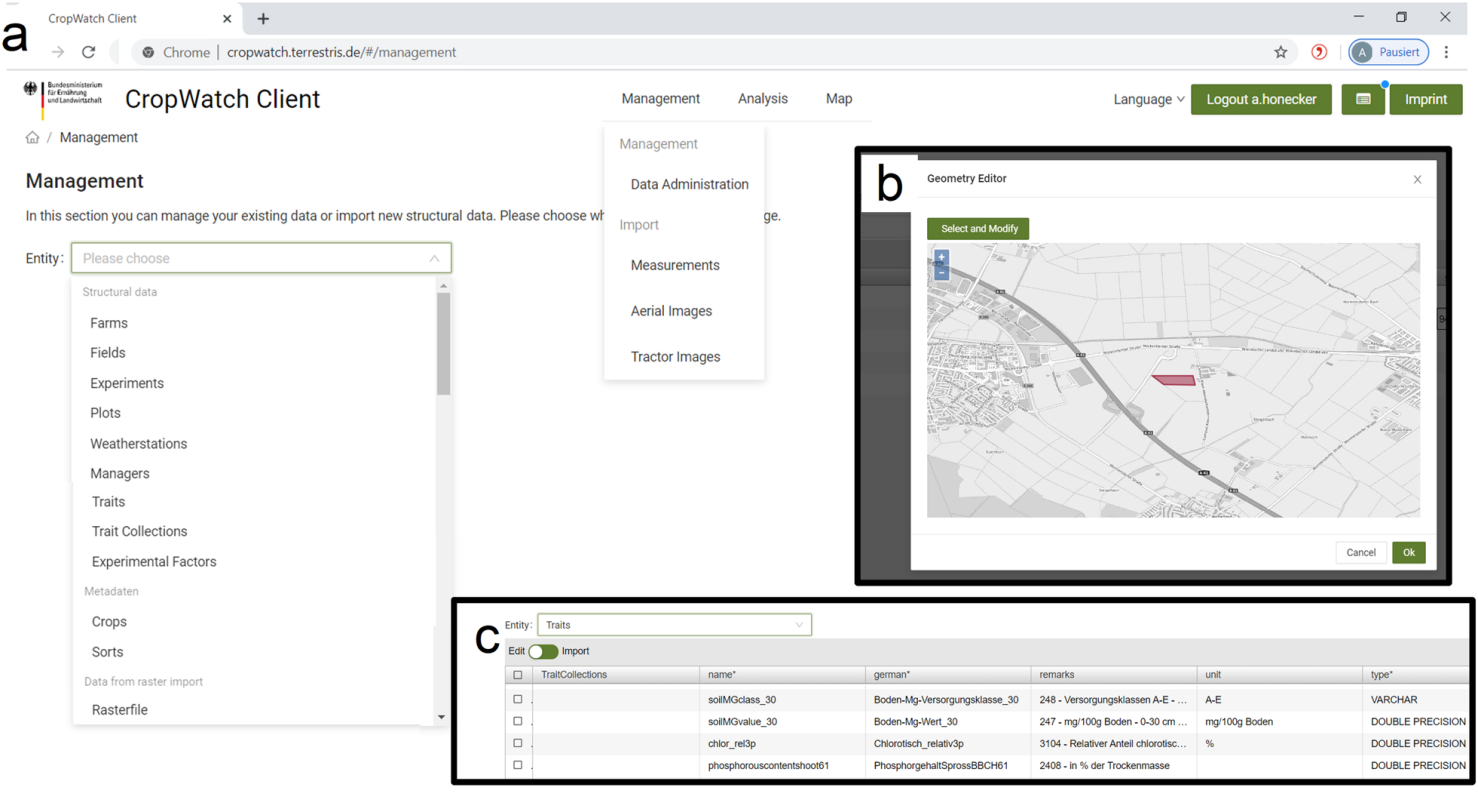
Management tool within the GUI. Composite of three screenshots of the management tool in the GUI. **a** Structural entities of the CPED. **b** Spatial information can be added either directly via coordinates, or drawn as geometries. **c** Phenotypic, agronomic and environmental traits can be created via the traits dialogue

The Management tool (Fig. 4) consists of an administration tab, where structural entities can be defined according to the data model (Fig. 4a).

They can be edited inside the management tool or in importable CSV-files. Mandatory identifiers are included to guarantee the valid structure of the database. Geometries are mandatory identifiers for all geographical structures and can be defined directly by importing their coordinates or drawn within the respective interface (Fig. 4b). Experimental factors within field trials are semantically connected and directly linked to the corresponding plots. This allows filtering or selecting data regarding their experimental factors, or factor levels. Phenotypic traits need to be defined by a representative name, type, unit and optional remarks. Thus, traits are properly documented and metadata is accessible (Fig. 4c). Similarly, non-numerical information, such as management actions or remarks, are referenced at the plot level and available in the system.

The combination of semantic and spatial references allows maximum customizability to the user. To ensure standardization of data within greater organizations, user-specific writing permissions can be defined. In research institutions, such users could be supervisors in academic field trials ensuring the usage of the “Findable, Accessible, Interoperable, Reusable” (FAIR) principles for data standardization [43]. Furthermore, metadata from crops, such as variety characteristics, can be added. This equally applies to all other types of data like soil, weather and management data.

With the Importer tool (Additional file 2) numerical data from field trials, as well as management and other non-numeric data can be imported (Additional file 2a). The data can be entered manually, or uploaded as CSV-files (Additional file 3), either as single traits or trait collections. The upload of image data is realized via upload masks (Additional file 2b) for collections of aerial images, single images from ground-based vehicles, or processed files such as orthophotos. Images without any spatial and temporal information need an additional semantic reference. This is achieved by the selection of experiment, date and the respective camera and image type.

**Fig. 5:**
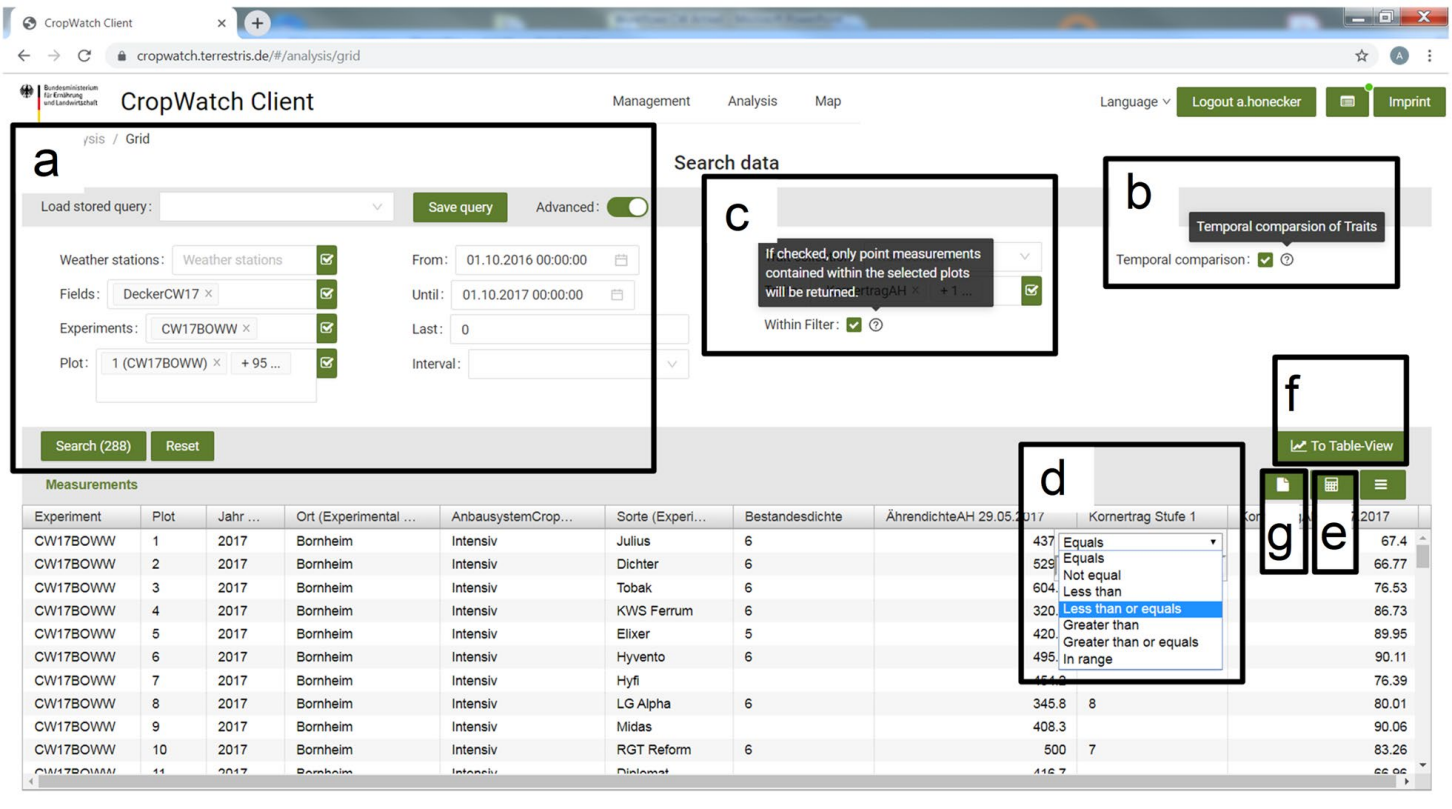
Data-handling tool within the GUI. **a** Measurement data can be selected according to semantic and temporal identifiers. Selection routines can be enhanced by (**b**) temporal comparison or (**c**) spatial selection to include only plot-related data. **d** Filtering options are integrated for detection of corrupted values or selection of single experimental factors or structural entities. **e** Descriptive statistics can be displayed and (**f**) charts can be displayed. **g** Selected data can directly be exported

With the Data-handling tool (Fig. 5) data can be selected by all structural entities and according to date and time (Fig. 5a). Selected data appears as a table below. Data can be displayed according to the timestamp, or temporally independent (Fig. 5b). This enables to compare phenotypic traits within time-series (see “Use case: weather-guided phenotyping”). If measurements are not semantically linked to an experiment or plot, but defined by geo-coordinates and timestamp, they can be displayed in the WebGIS without connection to an experiment. Such data can be a valuable source of information for trial planning (see “Use case: environmental information-based planning of field trials”). We used the geometries of the plots to filter for measurements with geo-coordinates. If the geo-coordinate of the measurement is located within the plot geometry, it is linked to the plot (Fig. 5c). Data can be filtered for outliers or obviously corrupted values (Fig. 5d). The advanced data selection methods within the Data-handling tool offer great flexibility to combine the relevant experimental units for further analysis. Due to its semantic connection, data can easily be prepared for advanced statistical analyzes in external statistical program packages.

Subsequently, an option to display simple descriptive statistics such as sum, mean, minimum or maximum values is included (Fig. 5e). Besides the table view, a charting module provides the opportunity to display data graphically (Fig. 5f). We implemented an export routine to support preparation of numerical data for advanced statistical analysis (Fig. 5g). Data can be exported as CSV-files, processed and re-imported as new traits.

**Fig. 6:**
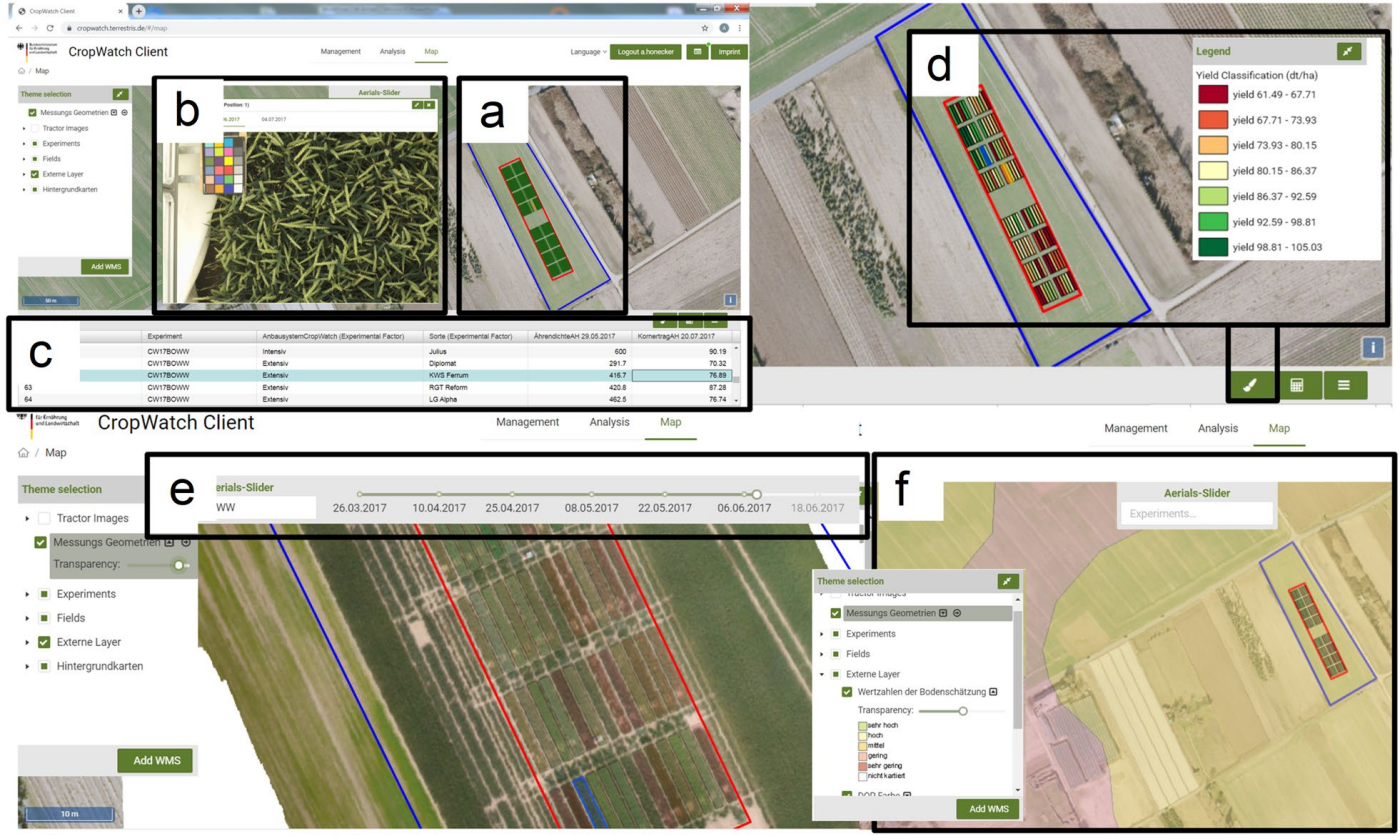
Mapping tool and WebGIS within the GUI. Composite of screenshots. **a** Geographical structures and **b** imported ground-based images can be displayed in the WebGIS. **c** Selected data is available in a table format. **d** Selected data is aligned to structural entities like plots and can be classified using the GeoStyler according to the trait value. **e** Processed aerial images like orthophotos can be displayed via the slider function. **f** Publicly available data like soil maps can be integrated via the use of WMS

With the Mapping tool (Fig. 6) the structural entities like fields, experiments, plots or weather stations can be displayed (Fig. 6a). Images and measurements independent of the semantic structure are displayed as point geometries, accessed and displayed on the map (Fig. 6b). Data selected in the handling tool can be viewed in a table format (Fig. 6c), with the respective geometry highlighted in the map (Fig. 6a, d). The implemented open source software tool GeoStyler [37] is capable of classifying the geometric structures. When investigating a certain trait within a field trial, plots will be classified by their trait value and colored accordingly (Fig. 6d). This becomes useful when searching for irregularities in the data, or spatial patterns within the trial (see use case “Data analysis”). Further geo-referenced data such as digital orthophotos can be displayed in time series by the slider function (Fig. 6e). Since WMS are integrated, soil maps, aerial images, or other publicly available mapped data^1^ can be imported and displayed in the DMIS (Fig. 6f).

### Application examples

The following use cases demonstrate new possibilities in the area of field trial planning, support of phenotyping practices and data evaluation, realizable with the DIMS.

### Use case: environmental information-based planning of field trials

Depending on the research question, especially in plant breeding, testing sites are selected by specified climatic and soil characteristics such as increased probability for drought stress. If testing sites show spatial heterogeneity, it is necessary to exclude these, or make use of the knowledge about the heterogeneity. This information could be utilized as co-variables in statistical models in order to getting statistically significant results.

**Fig. 7:**
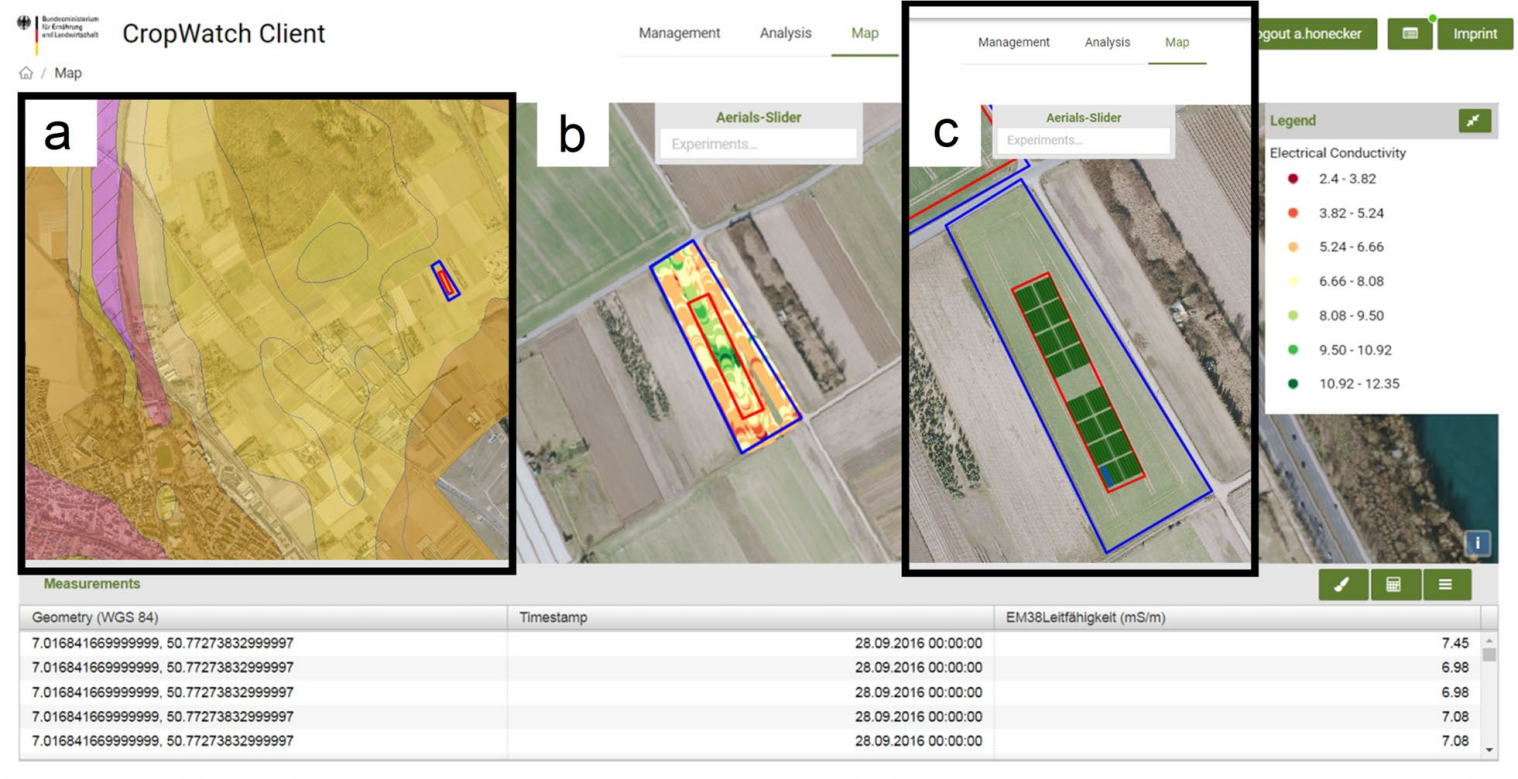
Use case: environmental information-based planning of field trials. Composite of screenshots from three different windows. **a** Initial selection of a suitable field location according to soil properties. **b** Detection of local heterogeneity by measuring apparent electrical conductivity. **c** Field trial design according to the information derived from the DMIS

The DMIS is able to support both, selection of the appropriate testing site and adjusting the spatial design according to local heterogeneity. By implementing official aerial images and soil maps via WMS (Fig. 7a) it was possible to screen the soils along the Rhine valley near Cologne, Germany, for sandy soils. Additional visualization of precipitation data from the corresponding official weather stations,^2^ allowed detecting a suitable trial site with a high potential for early summer drought (50°46′18.36″ N, 7°1′1.96″ E).

We used a sensor, measuring electrical conductivity [41] of the soil, point-wise and geo-referenced, to screen for spatial heterogeneity. Data was imported, classified and displayed (Fig. 7b), revealing a pattern of strong heterogeneity within the central area of the field. Subsequently, we excluded the specific area from our field trial (Fig. 7c). Thus, we utilized spatial information within the field trial design in order to achieve more homogeneous plant growth conditions.

The automated linking of data from soil measurements to the referring plot can be used as additional information, or as co-variables for a more precise and detailed statistical analysis of traits like crop yield.

### Use case: weather-guided phenotyping

**Fig. 8:**
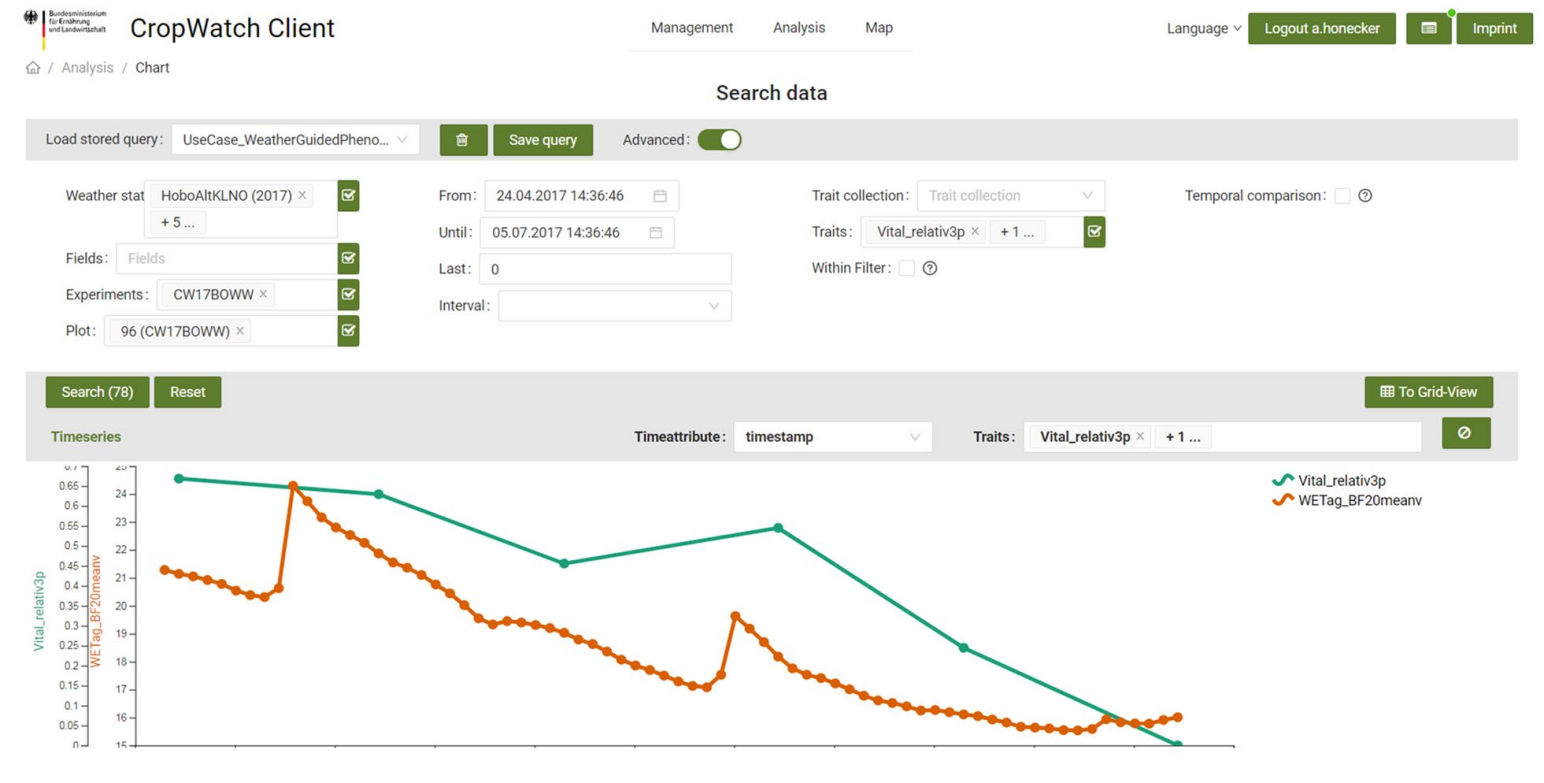
Use case–weather-guided phenotyping. Chart illustration of the development of relative soil water content (red) and corresponding relative vital leaf area of plants (blue) within a single trial plot. Vitality data is averaged by 3 images per plot each covering approx. 1 m^2^ canopy area

In the context of climate change, environmental stresses and their impact on yield formation become more and more important. To investigate related gene functions on the physiological reactions of the plants, it would be valuable to conduct the phenotypic measurements exactly when the environmental stress occurs. By monitoring weather parameters at the testing site, importing the data and using the charting module of the DMIS, it was possible to detect upcoming drought periods with decreasing soil moisture (Fig. 8). We captured geo-referenced images of the crop canopy from UAVs and ground-based platforms every 2 weeks during the vegetation period. Due to geo-referencing, images were automatically aligned to the respective plots during import. To derive information from the images, we designed image analysis routines to track the development of the relative fraction of green leaf area of the wheat canopy. With an upcoming shortage of available soil water, green leaf area decreases in parallel to soil moisture. As a result, screening of large breeding populations according to specific weather scenarios becomes possible. For visual control of the validity of the data and to verify observed effects the original images can directly be accessed and illustrated (Fig. 6b Mapping tool).

### Use case: data analysis for outlier or hot-spot detection

**Fig. 9:**
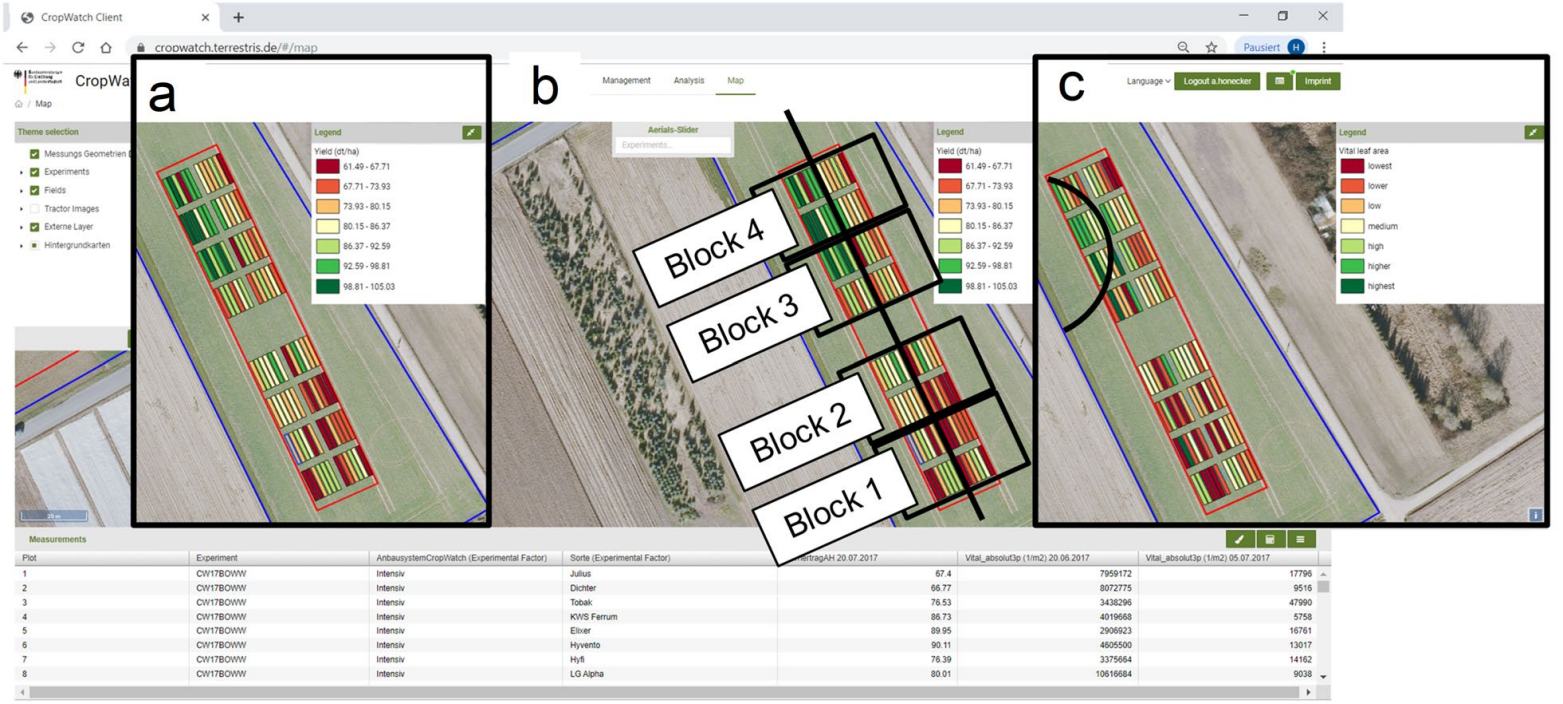
Use case: data analysis for outlier or hot-spot detection. Composite image with graphical remarks towards (**a**) identification of a spatial pattern of higher crop yield in the top left corner, **b** trial design. The obvious effect arises in blocks three and four. Initial ANOVA did not detect a significant block effect. Adding the geo-information by the DMIS enabled to adapt the statistical model accordingly. **c** Illustration of wheat green leaf area one month before harvest. The spatial pattern in block three and four is already visible. The black circular line indicates the irrigated area. Illustration of earlier measurements of green leaf area allowed the identification of the suspicious timeframe

We utilized the DMIS to combine measurement data and spatial information of the experimental plots assisted by the mapping functions (Fig. 9). By graphically visualizing the data in the WebGIS, we were able to revealing a pattern (Fig. 9a) that previously did not occur as an effect within our analysis of variance in the statistical evaluation of the fully randomized block design (Fig. 9b, 3rd and 4th block). Certain plots in the top left corner of the experiment showed significantly higher crop yields compared to all other plots (Fig. 9a, green = high yield, red = low yield). A comparison of the already described green leaf area data of these plots was visualized by images from one month before harvest. The data showed notably higher amounts of green leaf area in the respective plots (Fig. 9c). After further investigation, we were able to identify an irrigation event conducted by a circle sprinkler, passing in our experiment from the neighbor field and causing the irregularities. While statistically the pattern was masked by the block design, we were able to easily detect it by the mapping solution within the DMIS. By using this information as co-variables, the accuracy of the results of the field trial could be increased. We attached a video of this specific use case to illustrate the capabilities of the system (Additional file 4).

## Discussion

### System function validation

To prove the DMIS to function properly, we used data from the two-year wheat trial on two locations with 12 diverse wheat varieties and two cropping systems with four replications. Dealing with data from four environments, 96 variants, 384 plots and images from three points per plot and 10 time points per year resulted in 30 images and around 60 phenotypic traits per plot and year. The drone flights delivered approximately 300 aerial images for every field trial at eight time points per year for each of the four environments resulting in one orthophoto each. With the proposed DMIS, we were able to handle the large amount and the large variability of data successfully and to cover a real-world field trial scenario for the model crop winter wheat. However, the system is not specified on wheat and can easily be adjusted to other crops.

### Future prospects

The proposed data management and information system enables storing, managing, combining and visualizing all relevant data from the plant production process. 

Variety characteristics are included as described by the German Plant Variety Catalogue.^3^ To include these characteristics, we created an independent entity to store variety, or genotype specific information. Due to matching structural shapes, pedigree or further genotypic, such as marker information, could be integrated into the characteristics entity in a future version.

While data import works for all relevant data, automated routines for data import from external sources like official weather and soil service, or other sensors are not implemented at this stage.

Since the system is designed as an open source software solution and capable of connecting to standardized interfaces, automated data transfer and processing routines could be integrated into a future version. Furthermore, the open design of the DMIS allows linking of novel technologies like smart sensors, addressing internet-of-things approaches.

With the chart and mapping functions, we established a large toolbox for data visualization in the DMIS. Still, the functions could be further improved to display complex relationships and advanced charts.

Simple descriptive statistics can be computed and displayed. Since advanced data processing routines are not implemented in the current version, a module allowing to export the data in CSV-format was integrated into the system. It enables to choose the data of interest and export only the data needed for processing or advanced statistical analysis, thus reducing time for data selection. After processing, data can be re-imported as new traits and related to the respective structural entities.

We have already integrated publicly available data from soil via WMS or from climate. However, satellite data are a big future source of information. As an example, the data from the sentinel mission of the European Space Agency is now publicly available [44]. Since satellite data is geo-referenced, it might be integrated into the data structure with some effort in creating a work flow for getting, storing and presenting the data to serve as an additional, valuable source of agronomic information.

## Conclusions

With the proposed data management and information system, it is possible to handle phenotypic experimental field trials with crop plants. It focuses on linking all space and time dependent relevant processes of plant production, from weather and soil, to management, to growth and yield formation of the plants via geo-coordinates and timestamps. Therefore, a more precise interpretation of trial results in relation to environment and management is possible.

As potential users, breeders, extension specialists, official testing agencies and agricultural scientists can plan phenotypic experiments environment-specific, thus referring to soil heterogeneity. Raw data is stored in a structured and reliable manner. In particular, images from UAVs and pheno-mobiles can be integrated in the data management process. External processing of the raw images and raw data according to user-specific needs generates new informative phenotypic parameters that can then be stored, managed, combined and visualized to provide information for decision support. Traits can be individually defined by the user and trait descriptions must be given, thus supporting the FAIR guiding principles for data management. Scientists can use the system as a tool for the proper storage of experimental data, as demanded by the funding agencies of research projects. At research stations, the many field trials can be managed properly from year to year. In addition, farmers can use the system to handle their farm management using the structural entities farm, field and plot, skipping the field trial experiment entity.

Data availability, whenever needed, is the prerequisite for actions based on data. Since the DMIS is available via web-browsers, the integrated functions can be used both in field and in the office to derive space- and time-specific information.

Special weather scenarios, causing abiotic stresses, can be revealed and timed actions, such as phenotyping according to environmental conditions, can be planned. Since spatial patterns can be mapped due to spatial and temporal referencing of measurement data, adjusted management actions like precise hot-spot plant protection could be derived in the future.

As shown in the three use cases linking information from plant, space and time, the proposed data management and information system can help breeders, scientists, extension specialists and practicing farmers to manage their own “Big Data” and provide useful information from fields and field trials for decision support. Since resulting findings can be stored again, the DMIS enables the user to map the past to improve planning, managing and deciding in the future.

### Availability

The source code of the application “DMIS CropWatch” will be publicly available at the GitHub repository https://github.com/terrestris/.

A live version of the proposed DMIS is running with a sample dataset. We encourage to contact the authors for a test account or further information.

- Project name: CropWatch
- Project home page: https://github.com/terrestris/cropwatch/
- Archived version: backend v1.0.0, frontend v1.0.0, Git Hash 416ce4af8a167e0fc769d89d52cc6db81165e7c0
- Operating system(s): Platform independent
- Programming language: JavaScript
- Other requirements: node, docker
- License: BSD 2-Clause License
- Any restrictions to use by non-academics: –

## Supplementary information

**Additional file 1**: Detailed data structure in the data model of the DMIS. Entities of the level “Experiment” (experiment and experimental factors, plots, traits, measurements) are linked with entities of the level “Farm” (manager, farm, field, crop, management, weatherstation) and with the raster files. Future requirements can simply be mapped due to the dynamic adaptability of the model structure.

**Additional file 2:** Importer tool within the GUI. Composite of two screenshot parts in the Importer tool. **A** Measurement data can be imported to the CPED as single traits or previously defined as trait collections. Data input can be realized by manual input to the input mask or systematically as.csv-files. **B** Image data can be uploaded as aerial images with geographical reference or ground-based images (in our case only semantic references given) according to their data format and sensor. Semantic reference is established via connection to a structural entity as experiment and the reference timestamp.

**Additional file 3:** Example Input Data File. The file contains weather data from the vegetation period 2016/2017. Decimal separator must be “point” and field delimiter can be either “Semicolon”, “Tabulator”, “Space” or “Comma” and must be adjusted accordingly during the import procedure in the Importer tool.

**Additional file 4:** Screen recording of the DMIS. The video illustrates the capabilities of the DMIS via the example use case “Data analysis for outlier or hot-spot detection”. All necessary steps from data selection to mapping within the WebGIS are displayed.

## Data Availability

The software generated is available at https://github.com/terrestris/cropwatch/. A live version of the proposed DMIS is running with a sample dataset. It can be accessed upon request.

## Abbreviations

UAV: Unmanned aerial vehicle
PDA: Personal digital assistant
geo: Geographic
GPS: Global positioning system
DMIS: Data management and information system
GUI: Graphical user interface
CPED: Central processing and exchange database
WebGIS: Web browser based Geographical Information System
WMS: Web mapping service
FAIR: Findable, accessible, interoperable, reusable

## Footnotes

1 IMA GDI.NRW, Geobasis NRW, Muffendorfer Str. 19-21, 53177 Bonn (https://www.opengeodata.nrw.de/produkte/geobasis/).

2 German Weather Service, Frankfurter Straße 135, 63067 Offenbach, Germany (https://opendata.dwd.de/climate_environment/CDC/).

3 https://www.bundessortenamt.de/bsa/en/variety-testing/descriptive-variety-lists/descriptive-variety-lists-published-by-the-federal-plant-variety-office/.
