# Supplementary figures and images for: Plant, space and time - linked together in an integrative and scalable data management system for phenomic approaches in agronomic field trials
Source: Plant Methods. 2020 Apr 21;16:55. doi: 10.1186/s13007-020-00596-3 (PMC7171732; doi:10.1186/s13007-020-00596-3)

## Slide 1
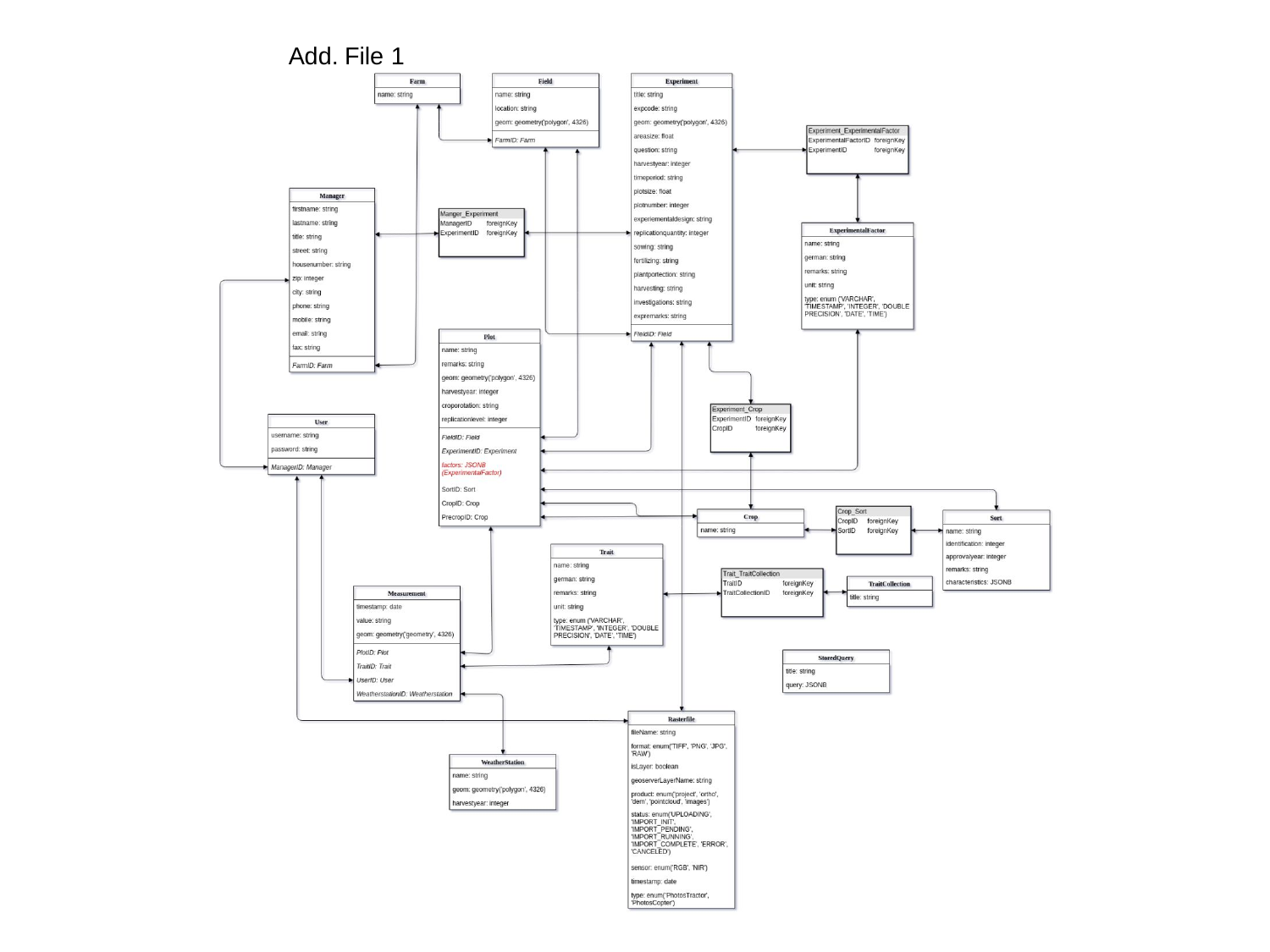

Supplement: Supplementary file 1 — Additional file 1: Detailed data structure in the data model of the DMIS. Entities of the level “Experiment” (experiment and experimental factors, plots, traits, measurements) are linked with entities of the level “Farm” (manager, farm, field, crop, management, weatherstation) and with the raster files. Future requirements can simply be mapped due to the dynamic adaptability of the model structure. [file 13007_2020_596_MOESM1_ESM.pptx]

## Slide 1
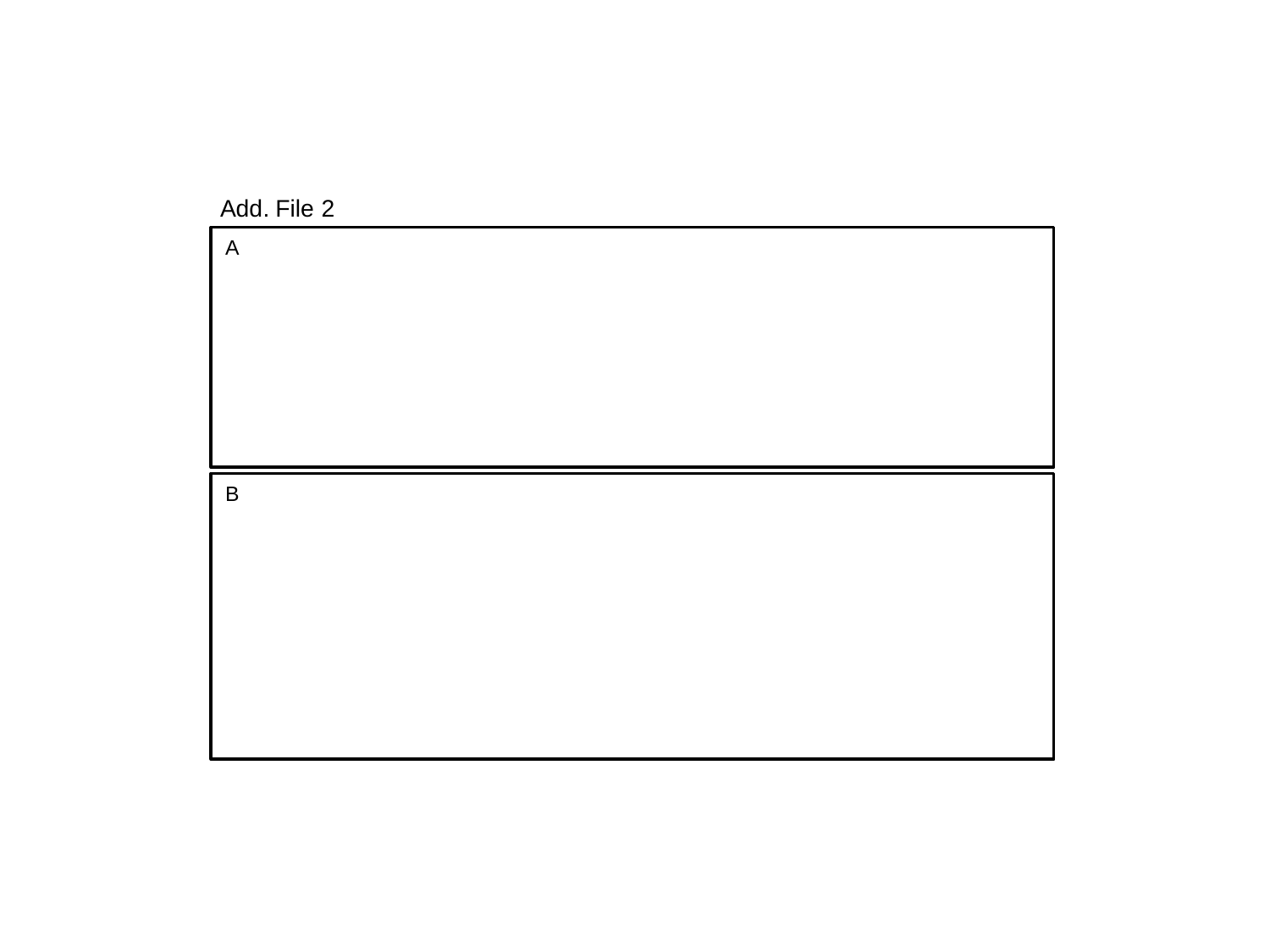

Supplement: Supplementary file 2 — Additional file 2: Importer tool within the GUI. Composite of two screenshot parts in the Importer tool. A Measurement data can be imported to the CPED as single traits or previously defined as trait collections. Data input can be realized by manual input to the input mask or systematically as.csv-files. B Image data can be uploaded as aerial images with geographical reference or ground-based images (in our case only semantic references given) according to their data format and sensor. Semantic reference is established via connection to a structural entity as experiment and the reference timestamp. [file 13007_2020_596_MOESM2_ESM.pptx]
